# Equivalent Properties of Transition Layer Based on Element Distribution in Laser Bending of 304 Stainless Steel/Q235 Carbon Steel Laminated Plate

**DOI:** 10.3390/ma11112326

**Published:** 2018-11-19

**Authors:** Zihui Li, Xuyue Wang, Yonghao Luo

**Affiliations:** School of Mechanical Engineering, Dalian University of Technology, Dalian 116023, China; lizihui@mail.dlut.edu.cn (Z.L.); wbzzd@dlut.edu.cn (X.W.)

**Keywords:** laser bending, equivalent property, laminated plate, transition layer, element distribution, finite element model

## Abstract

Compared with the single-component metal plate, there is a special transition layer on the joint interface between two kinds of materials in the stainless steel-carbon steel laminated plate (SCLP). In order to describe the finite element model of laser bending accurately, it is of great significance to determine material properties of the transition layer. Based on the element distribution, an equivalent method is adopted to calculate thermal conductivity, thermal expansion coefficient, elastic modulus, density, Poisson’s ratio, and specific heat capacity of transition layer. The electron probe experiments show that the transition layer is formed by interfacial element diffusion with thickness of 7 μm. Besides, the volume fraction of stainless steel (46.63%) and carbon steel (53.37%) in the transition layer is tested by energy dispersive spectrometer, respectively. Through the equivalent method, a laser bending model of SCLP is simulated by ANSYS software to predict the bending angle under different parameters. The experimental verification shows that the maximum of bending angle errors is 3.74%, which is lower than the maximum 4.93% of errors calculated by the mean value method. The analysis verifies that the laser bending model is feasible and contributes to improving the accuracy of modeling SCLP in the laser bending process.

## 1. Introduction

**Figure 1:**
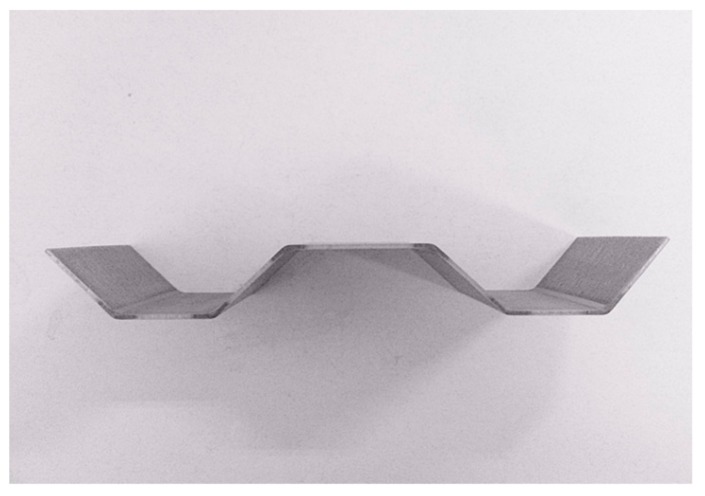
Two-period corrugated bulkhead sample.

Because of the high temperature resistance and corrosion resistance of 304 stainless steel, and high strength and high thermal conductivity of Q235 carbon steel [1], SCLP has broad application prospects in corrugated bulkhead of flying machine and deep-sea scuba machine, as shown in Figure 1. As a new kind of composite material, SCLP has a transition layer between the matrix (carbon steel) and cladding (stainless steel), formed by physical contact, chemical reaction and element diffusion. Shen et al. [2] prepared the composite material of 304 stainless steel-reinforced Q235 carbon steel by a modified hot-rolling process. It was found that a metallurgical bonding was formed between the stainless steel layer and the carbon steel substrate. Dhib et al. [3] pointed out that the hot-roll bonding process caused the formation of a thin diffusion layer with decarburized ferrite zone of the parent metal and carburized austenite zone of the clad layer. Zhang et al. [4] investigated the behaviors and mechanisms of diffusion welding between 304 stainless steel and pure Ni based on molecular dynamics simulation, and found that atoms diffused into the opposite side and diffusion distance increased with rise in temperature.

A corrugated bulkhead contains a large number of bending structures. In a bending process, laser bending does not require molds, contacts, or external forces. As a newly developed flexible forming technology, it is suitable for forming plates. Because of the complexity of material properties and the interaction between laser and material, domestic and foreign scholars focus on accurate modeling in laser bending simulation. For this reason, many scholars have devoted a lot of work to calculate composite material properties. Liu et al. [5] predicted the bending angle of SiC reinforced aluminum matrix composite based on the Vollertsen’s model in laser forming by calculating equivalent properties. Gou et al. [6] calculated thermal expansion properties of textile-reinforced composites with certain structure symmetries by a unit cell model. Mishurova et al. [7] calculated equivalent elastic properties of polymer-fiber reinforced concrete in the framework of the non-interaction approximation, the Mori-Tanaka-Benveniste scheme, and the Maxwell scheme. During the laser bending process, the transition layer is an extremely important component of composite plate and its properties are key factors affecting load transfer, stress distribution and deformation; the transition layer of laminated composite plate should be fully considered in terms of modeling. Because there are no specific properties of the transition layer in existing manuals, scholars use a variety of methods to unify the calculation of material properties for the transition layer. For a bilayer material system including an Al 6061 metal layer and a SiC ceramic layer, Shen et al. [8] analyzed the temperature and stress fields during the laser-forming process, but did not consider the combination mechanisms of the joint surface and introduction into the model. Song et al. [9] calculated the physical properties of the transition layer by mean value method, and simulated deformation behaviors of the SCLP model. At present, material properties of the transition layer are mainly calculated by the mean value method. However, the mean value method cannot reflect the heterogeneity of material distribution in the transition layer, which is a key factor affecting the angle of laser bending. Therefore, it is particularly important to accurately describe the dimensions and material properties of the transition layer by an equivalent method considering the admixture of 304 stainless steel and Q235 carbon steel.

In this work, the element distribution in the SCLP and the transition layer thickness are obtained by electron probe experiments and the element content in the SCLP is measured by energy dispersive spectrometer. Then, the volume fraction of stainless steel and carbon steel in the transition layer are calculated combining the element content in the SCLP. On account of the volume fraction and the element distribution of the transition layer, the calculation of the equivalent properties of the transition layer (including thermal conductivity, thermal expansion coefficient, elastic modulus, density, Poisson’s ratio and specific heat capacity) is carried out by the effective medium theory, Turner formula, Mori-Tanaka method and mixture law. Furthermore, a finite element model of laser bending is developed based on the equivalent properties of the transition layer. The bending angle of laser processing is predicted by the simulation of laser loading and material deformation, which provides a theoretical and experimental basis for laser bending of SCLP.

## 2. Materials and Methods

### 2.1. Element Distribution of Transition Layer

**Figure 2:**
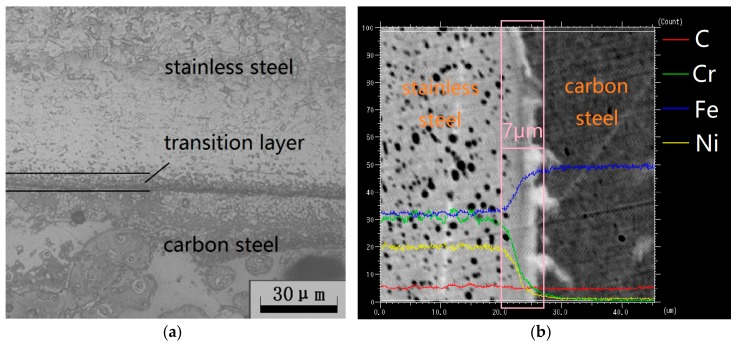
Metallographic microscope observation of the SCLP: (**a**) Metallographic micrograph; (**b**) electron probe microanalysis.

The SCLP is prepared by combining the matrix (carbon steel) and cladding layer (stainless steel) with special processes. Firstly, metallurgical bonding of the matrix and cladding layer is realized by explosion. Then, the thickness of the SCLP is controlled by hot rolling and cold rolling. Finally, surface finishing is necessary to improve overall quality of the SCLP. Figure 2 is the micrograph of the interface obtained by the metallographic microscope and the electron probe.

As shown in Figure 2a, there is a dark ribbon zone with a width of approximately 7 μm near the joint surface of the SCLP, which is the transition layer between the stainless steel and the carbon steel. According to the element count intensity shown in Figure 2b, Fe content in the carbon steel layer is much higher than that in the stainless steel layer, but it does not contain Ni or Cr. Therefore, the diffusion direction of Fe is from carbon steel to stainless steel, and the diffusion direction of Ni and Cr is the opposite. Moreover, because the difference of carbon content between the two materials is not significant, no obvious diffusion phenomenon of C element is observed in the transition layer.

**Figure 3:**
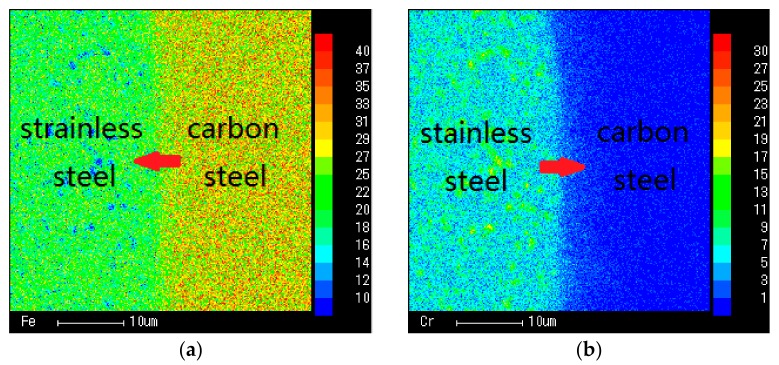

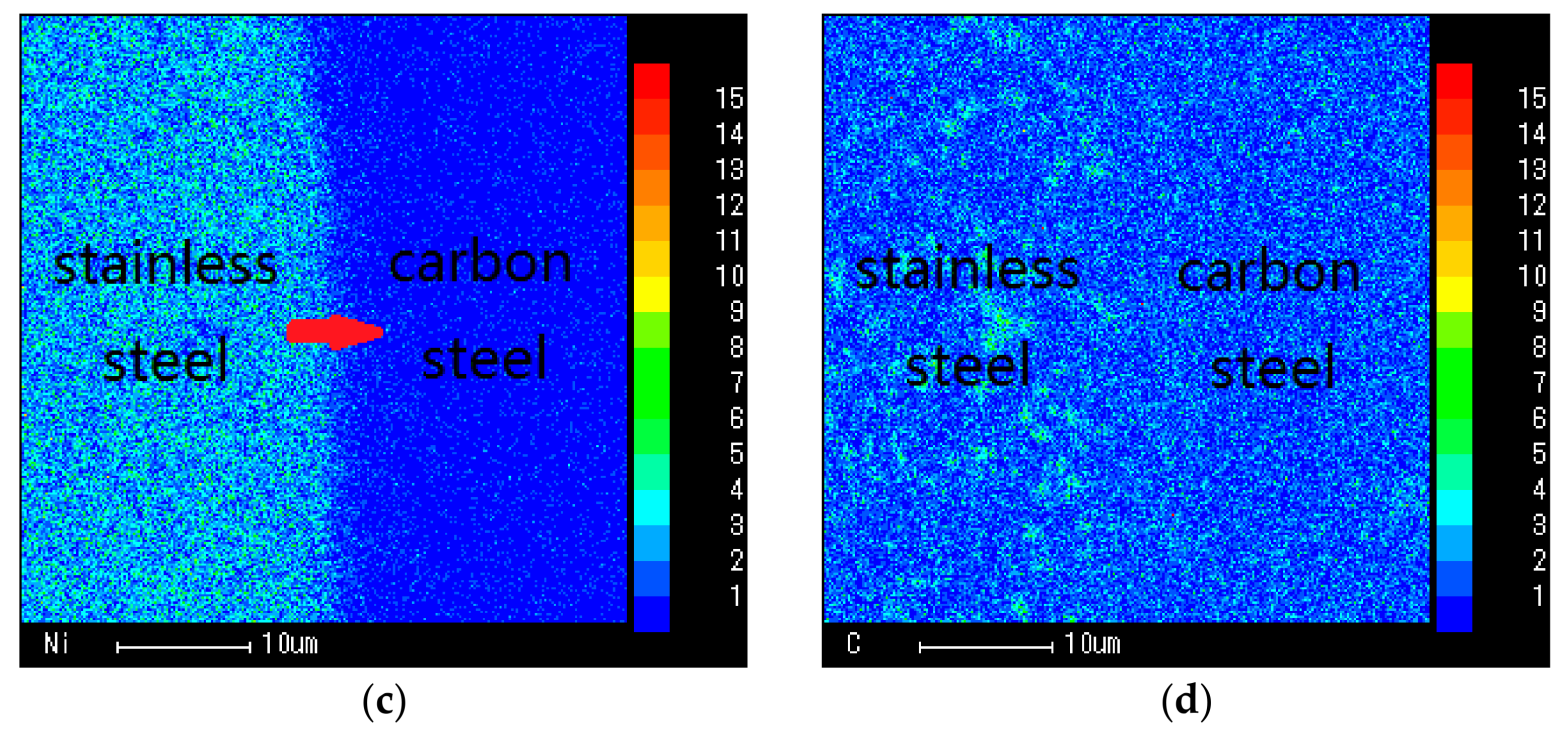
Diffusion and distribution of elements in the transition layer: (**a**) Diffusion and distribution of Fe; (**b**) Diffusion and distribution of Cr; (**c**) Diffusion and distribution of Ni; (**d**) Diffusion and distribution of C.

Figure 3 is the result of element diffusion in the transition layer. The distribution of Fe, Cr, Ni and C elements verifies the diffusion direction due to the concentration gradient. As per the element content, the transition layer is regarded as a heterogeneous composite with the characteristic of a normalized parameter, and the equivalent model of the transition layer is established by combining the interparticle interaction in the material; thus, the angle of the laser bending SCLP is predicted.

### 2.2. Laser Bending Experiment and Bending Angle Measurement

**Figure 4:**
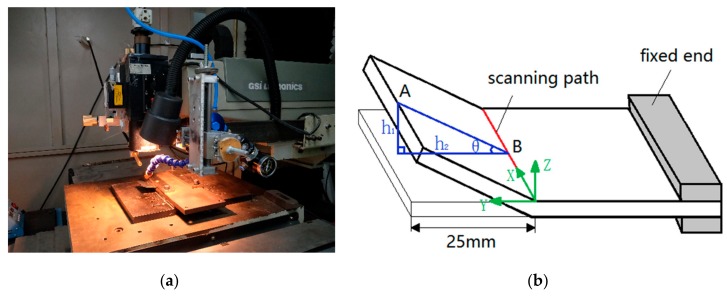
Experimental devices of laser bending: (**a**) Laser bending system; (**b**) Schematic diagram of laser bending.

The laser bending experiment is performed on a LMT-5040 precise numerical control machine (Beijing Precision Machinery Manufacturer, Beijing, China) using JK701H Nd:YAG pulsed laser (with the wavelength of 1.064 μm) (GSI Lumonics, London, UK) shown in Figure 4a. The fixation of the SCLP (with dimension of 60 mm × 50 mm × 1 mm) is single-end clamped with the free end 25 mm away from the scanning path shown in Figure 4b. In order to determine the bending angle, a tangent calculation method is used by the measurement of trilinear coordinates measuring instrument (with the precision of 1 μm) (ZEISS, Oberkochen, Germany) as follows:

*β* = arctan(*h*_1_/*h*_2_) (1)

where *β* is the bending angle of the SCLP, *h*_1_ is the rising height of the free end, and *h*_2_ is the projection of AB in the Y-direction.

The experimental devices and processing parameters such as power, scanning speed, defocus distance and scanning time are shown in Figure 4 and Table 1, respectively.

## 3. Simulations and Analysis

Because the transition layer is a region formed by inter-diffusion of elements in stainless steel and carbon steel, its properties and constituent elements are different from those of both sides. There are no specific transition layer properties in the existing manuals, so the transition layer needs to be theoretically calculated to improve modeling accuracy using the finite element method. According to the assumptions, the transition layer is considered to consist of stainless steel with volume fraction of *V*_1_ and carbon steel with volume fraction of *V*_2_. According to the transition layer bonding mechanism, the corresponding theoretical formula is selected to calculate the equivalent properties, that is, the material properties of the transition layer.

### 3.1. Calculation of Equivalent Properties of Transition Layer

#### 3.1.1. Equivalent Thermal Conductivity

Zhou et al. pointed out that due to the differing content of diffused materials, the calculation methods were diverse in the equivalent thermal conductivity calculation model. In this work, the content of stainless steel and carbon steel in the transition layer of the SCLP is almost equal. The heterogeneous continuous phase or dispersed phase can be calculated by the equivalent medium theory. The calculation process of the equivalent thermal conductivity of the transition layer can be expressed as follows [10]:

(2) $$k_{c}=\frac{1}{4}\left[k_{1}\left(3V_{1}-1\right)+k_{2}\left(3V_{2}-1\right)+\sqrt{{\left[k_{1}\left(3V_{1}-1\right)+k_{2}\left(3V_{2}-1\right)\right]}^{2}+8k_{1}k_{2}}\right]$$

where *k_c_* is the thermal conductivity of transition layer, *k*_1_ and *k*_2_ are the thermal conductivity of stainless steel and carbon steel, respectively; *V*_1_ and *V*_2_ are the volume fraction of stainless steel and carbon steel, respectively.

#### 3.1.2. Equivalent Thermal Expansion Coefficient

Makarian et al. [11] developed a predictive model and verified the equivalent thermal expansion coefficient of particulate composites as a function of the thermo mechanical and geometrical properties of the individual constituents. Turner [12] considered that an internal stress existed in a mixture such that the stresses were nowhere sufficient to disrupt the material; the sum of the internal forces could be equated to zero and an expression for thermal expansion coefficient of the mixture was obtained. According to the Turner model, thermal expansion coefficient of transition layer is given by:

(3) $$\alpha_{c}=\left(\alpha_{1}K_{1}V_{1}+\alpha_{2}K_{2}V_{2}\right)/\left(K_{1}V_{1}+K_{2}V_{2}\right)$$

where *α_c_* is the thermal expansion coefficient of transition layer, *α*_1_ and *α*_2_ are the thermal expansion coefficient of stainless steel and carbon steel, respectively, *K*_1_ and *K*_2_ are the bulk modulus of stainless steel and carbon steel, respectively.

According to the formula of bulk modulus, *K*_1_ and *K*_2_ can be expressed as follows:

(4) $$K_{1}=E_{1}/3(1-2\lambda_{1})$$

(5) $$K_{2}=E_{2}/3(1-2\lambda_{2})$$

where *E*_1_ and *E*_2_ are the elasticity modulus of stainless steel and carbon steel, respectively; and *λ*_1_ and *λ*_2_ are the Poisson’s ratio of stainless steel and carbon steel, respectively. The Equations (4) and (5) are substituted into Equation (3) to obtain the equivalent thermal expansion coefficient as follows:

(6) $$\alpha_{c}=\left[\alpha_{1}E_{1}V_{1}(1-2\lambda_{2})+\alpha_{2}E_{2}V_{2}(1-2\lambda_{1})\right]/\left[E_{1}V_{1}(1-2\lambda_{2})+E_{2}V_{2}(1-2\lambda_{1})\right]\;$$

#### 3.1.3. Equivalent Elasticity Modulus

Mori and Tanaka [13] provided simple tools for the evaluation of the equivalent elasticity modulus of transition layer. The equivalent modulus method for non-homogeneous material assumed that average internal stress was uniform and took into account the effects of the interaction among the inclusions. The relationship about the equivalent elastic modulus of transition layer is given by using the mean field theory as follows:

(7) $$K_{c}/K_{2}=1+V_{1}(K_{1}-K_{2})/\left[K_{2}+\alpha (1-V_{1})(K_{1}-K_{2})\right]\;$$

where *K_c_* is the bulk modulus of transition layer, *α* is the parameter that replaces variables. They are expressed as follows:

(8) $$K_{c}=E_{c}/3(1-2\lambda_{c})\;$$

(9) $$\alpha =\left(1+\lambda_{2}\right)/3\left(1-\lambda_{2}\right)$$

Therefore, the equivalent elasticity modulus for the transition layer can be obtained by the simultaneous formulas of Equations (7)–(9):

(10) $$E_{c}=3K_{2}(1-2\lambda_{c})+3(1-2\lambda_{c})K_{2}V_{1}(K_{1}-K_{2})/\left[K_{2}+\alpha (1-V_{1})(K_{1}-K_{2})\right]\;$$

where *E_c_* is the equivalent elasticity modulus for the transition layer, and *λ_c_* is the Poisson’s ratio of the transition layer.

#### 3.1.4. Other Equivalent Properties

In other aspects of the equivalent properties such as density, Poisson’s ratio and specific heat capacity are calculated by the law of mixing of composite materials:

(11) $$\rho_{c}=\rho_{1}V_{1}+\rho_{2}V_{2}$$

(12) $$\lambda_{c}=\lambda_{1}V_{1}+\lambda_{2}V_{2}\;$$

where *ρ_c_* is the equivalent density of transition layer, *ρ*_1_ and *ρ*_2_ are the density of stainless steel and carbon steel, respectively. Similarly, the specific heat of transition layer is given by:

(13) $$C_{c}=\left(\rho_{1}V_{1}C_{1}+\rho_{2}V_{2}C_{2}\right)/\left(\rho_{1}V_{1}+\rho_{2}V_{2}\right)\;$$

where *C_c_* is the equivalent specific heat capacity of the transition layer; and *C*_1_ and *C*_2_ are the specific heat capacity of stainless steel and carbon steel, respectively.

### 3.2. Calculation Results of Materials Equivalent Properties

As shown in Table 2, the composition and content of elements are detected by energy dispersive spectrometer of a scanning electron microscopic for each layer of SCLP.

According to calculations, the volume fraction of Fe, Cr, Ni and C diffused from the stainless steel layer are 46.06%, 48.55%, 53.35% and 40.00%, respectively, and from carbon steel are 53.94%, 51.45%, 46.65% and 60.00%, respectively. Because of the high content of Fe, the diffusion of Fe plays an important role in the formation of the transition layers. Therefore, the proportion of carbon steel in transition layer is slightly larger than that of stainless steel. Based on the weighted average method, the material of transition layer is composed of 46.63% stainless steel and 53.37% carbon steel. This volume fraction will be used to calculate the equivalent material properties in the transition layer.

**Figure 5:**
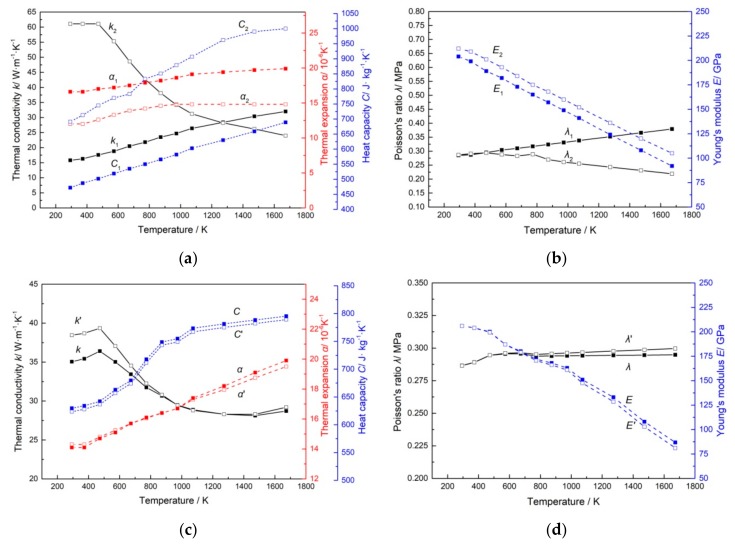
Material properties of the SCLP: (**a**) Thermodynamic properties of stainless steel and carbon steel; (**b**) Mechanical properties of stainless steel and carbon steel; **(c**) Comparison of thermodynamic properties of transition layer between the two calculation methods; (**d**) Comparison of mechanical properties of transition layer between the two calculation methods.

According to the material volume fraction of the transition layer, the calculation results of equivalent properties are shown at room temperature in Table 3. At the same time, temperature parameters are taken into consideration. Figure 5a,b show thermodynamic and mechanical properties of stainless steel (X_1_) and carbon steel (X_2_) in the manual. Based on the calculation of MATLAB software, Figure 5c,d show a comparison of the thermodynamic and mechanical properties between the equivalent method (X) and the mean value method (X’).

In the mean value method, the transition layer is regarded as a homogeneous mixture of stainless steel and carbon steel with equal proportion. However, according to the element distribution, it is known that there exists an inhomogeneous state in the transition layer. Parrott et al. [14] pointed out that the mean value method of the thermal conductivities for two components was mathematically unsound and tended to overestimate the conductivity of the system. Therefore, the thermal conductivity calculated by the equivalent method is lower than the mean value method. For heterogeneous materials, the equivalent calculation of the transition layer takes into account the interaction between the internal particles, which is more suitable for predicting the thermal expansion coefficient of the composite than that of the mean value method. Moreover, the mean value method, without considering the proportions of the two materials in the transition layer, has a larger error in calculation of specific heat capacity due to the obvious difference between stainless steel and carbon steel. Other properties such as elastic modulus, density and Poisson’s ratio of stainless steel and carbon steel have relatively consistent tendency with temperature change. The approximation of the two materials makes the results of these properties obtained by the mean value method and the equivalent method basically the same.

### 3.3. Finite Element Model of SCLP Considering Transition Layer

**Figure 6:**
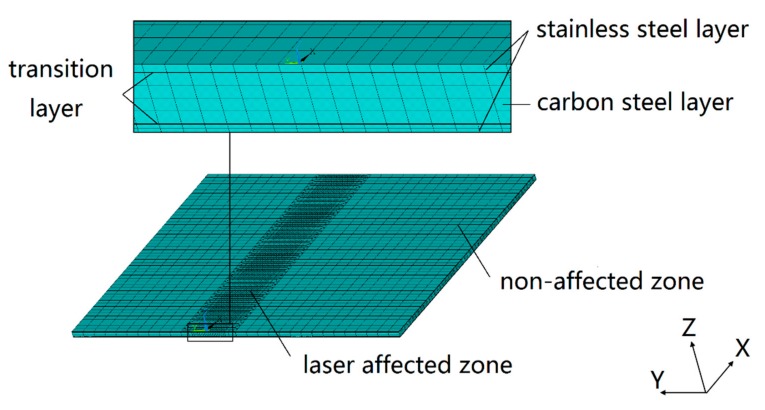
FEM grid partition profile of the SCLP.

According to the transition layer properties obtained by the equivalent method, a 1:1 geometric model of the SCLP is established based on the actual work piece. In order to meet the requirements of manufacturing standards, the thickness of stainless steel, transition layer and carbon steel layer in the SCLP model with a size of 60 mm × 50 mm × 1 mm is 120 μm, 7 μm and 746 μm, respectively. In the ANSYS simulation of laser bending SCLP, mesh density has an important influence on the accuracy and efficiency of calculation. Figure 6 shows the FEM grid partition profile of the SCLP in different directions. Due to the large temperature and stress gradient in a laser-affected zone with width of approximate 6 mm, the grid of the XOY plane is divided by the finer specification of 1/3 mm × 1/3 mm. In contrast, the temperature and stress field in the non-affected zone are relatively uniform, so the grid is chosen as the looser 1 × 1 mm specification. In the Z axis direction, the thickness of stainless steel, transition layer and carbon steel layer is 120 μm, 7 μm and 746 μm with different grid division, respectively (stainless steel 2 × 60 μm, transition layer 1 × 7 μm, and carbon layer 4 × 186.5 μm). The model consists of 60,450 elements and 61,724 modes. Eight-node three-dimensional element is used for both thermal and mechanical analyses.

The numerical simulation of laser bending SCLP is a nonlinear transient thermal elastoplastic deformation process by ANSYS software (ANSYS 14.5, ANSYS, Inc., Canonsburg, PA, USA). The Newton-Raphson method is used for iterative solution. In order to speed up the convergence, the large deformation option and the automatic step option are turned on. The algorithm used in the ANSYS software is an implicit algorithm in the direct integration.

The pulsed laser moves with uniform speed along the scanning line to heat the upper stainless steel surface. In this paper, assuming that the single-pulse laser energy is a dynamic heat source that obeys the Gaussian distribution, the power density distribution of pulsed laser can be expressed as:

(14) $$I(x,y)=AI_{0}f(x,y)g(t)$$

where *A* is the absorption coefficient (about 0.25), *I*_0_ is the power density at the laser center, *f*(*x*,*y*) is the spatial distribution of pulsed laser, *g*(*t*) is the time distribution of pulsed laser. For the fundamental mode Gauss beam, *f*(*x*,*y*) and *g*(*t*) can be expressed as:

(15) $$f(x,y)=\exp\left\{-2\left[{\left(x-x_{0}\right)}^{2}+{\left(y-y_{0}\right)}^{2}\right]/r_{0}^{2}\right\}$$

(16) $$g(t)=\left\{ \begin{array}{ll} 1 & 0\leq t\leq t_{p}\\ 0 & t_{p}<t\leq 1/f \end{array}\right.$$

where *x*_0_ and *y*_0_ are the coordinates of laser spot center, *x* and *y* are the current coordinates within the laser spot area, *r*_0_ is the spot radius when light intensity drops to 1/*e* center intensity, *t* is the time, *t_p_* is the pulse width (2 ms), and *f* is the laser frequency (40 Hz).

### 3.4. Finite Element Equations of Heat Conduction

Assuming that the material of each layer is uniform, continuous and isotropic, and the properties change with temperature, the transient temperature field variable *T*(*x*,*y*,*z*,*t*) satisfies Fourier’s law and energy conservation law in a Cartesian coordinate system. When laser heats the plate, the three-dimensional transient heat conduction equation is:

(17) $$\lambda_{j}\left(\frac{\partial^{2}T_{j}}{\partial x^{2}}+\frac{\partial^{2}T_{j}}{\partial y^{2}}+\frac{\partial^{2}T_{j}}{\partial z^{2}}\right)=\rho_{j}c_{j}\overset{\cdot }{T_{j}}$$

where *T_j_* is the instantaneous temperature distribution function of the *j*-th layer, *ρ_j_*, *c_j_*, *λ_j_* are density, specific heat and thermal conductivity of the *j*-th layer, respectively, *j* = 1, 2, …, *n*. Assuming that the temperature and heat flow of different materials at the junction are continuous, the equations can be written as:

(18) $$T_{j}(x,y,z,t)=T_{j+1}(x,y,z,t)\;$$

(19) $${-\lambda_{j}\frac{\partial T_{j}}{\partial z}|}_{z=h_{j}}={-\lambda_{j+1}\frac{\partial T_{j+1}}{\partial z}|}_{z=h_{j}}$$

There is radiation and convection between the SCLP and the ambient environment, and the boundary condition can be written as follows:

(20) $$-k\cdot \partial T/\partial n=h_{e}(T-T_{0})\;$$

where *k* is thermal conductivity, and *h_e_* is equivalent convective heat transfer coefficient.

(20) $$h_{e}=h_{c}+h_{r}\;$$

(21) $$h_{r}=\varepsilon \sigma (T_{w}+T_{0})(T_{w}^{2}+T_{0}^{2})$$

where *h_c_* is convection heat transfer coefficient, *h_r_* is radiative heat transfer coefficient, *ε* is emissivity coefficient, *σ* is Stefan-Boltzmann constant, and *T_w_* is the boundary temperature of the workpiece. Refer to the range of equivalent convective heat transfer coefficient in different environments, the value is 30 W·m^−2^·K^−1^. *T* is the temperature of the workpiece.

Initial condition:

(23) $${T|}_{t=0}=T_{0}\;$$

where *T*_0_ is the initial temperature considered as 293 K.

Since the temperature, heat flow rate, thermal boundary conditions and internal energy of the SCLP change significantly with time during the laser bending process, the Equation (17) is solved by the Galerkin method. The heat balance matrix equation of the SCLP is obtained in nonlinear thermal analysis as follows:

(22) $$\left[C(T)\right]\left\{\overset{\cdot }{T}(t)\right\}+\left[K(T)\right]\left\{T(t)\right\}=\left\{Q(t)\right\}$$

where [*C*(*T*)] is the matrix of special heat capacity, {$\dot{T}$(*t*)} is the derivative of the node temperature with respect to time, [*K*(*T*)] is the matrix of heat conduction, {*T*(*t*)} is the vector of node temperature, and {*Q*(*t*)} is the vector of the node’s heat flow rate.

Using the backward difference method to discretize Equation (24) in time, the differential equations are linearized to obtain the control equation of the transient temperature field as follows:

(23) $$\left(\left[K(T)\right]+\frac{\left[C(T)\right]}{\Delta t}\right){\left\{T\right\}}_{t}={\left\{Q\right\}}_{t}+\frac{\left[C(T)\right]}{\Delta t}{\left\{T\right\}}_{t-\Delta t}\;$$

### 3.5. Finite Element Equations of Thermal Elastic-Plasticity

Incremental theory is used to establish the relationship between thermal elastoplastic stress increment and strain increment for the SCLP. In the laser bending process, the deformation zone includes two parts of elastic deformation zone and plastic deformation zone. In the elastic zone, the total strain increment can be expressed as:

(24) $$\{d\varepsilon \}={\left\{d\varepsilon \right\}}_{e}+{\left\{d\varepsilon \right\}}_{T}\;$$

where {*dε*}*_e_* is elastic strain increment, and {*dε*}*_T_* is thermal strain increment caused by temperature change.

In the plastic zone, the total strain increment can be expressed as:

(25) $$\{d\varepsilon \}={\left\{d\varepsilon \right\}}_{e}+{\left\{d\varepsilon \right\}}_{p}+{\left\{d\varepsilon \right\}}_{T}\;$$

where {*dε*}*_p_* is plastic strain increment. During hot deformation, the stress-strain relationship can be expressed as:

(26) {dσ}=[D]{dε}−{C}dT 

In the elastic zone, [*D*] = [*D*]*_e_*, [*D*]*_e_* is elastic matrix, [*C*] = [*C*]*_e_*. In the plastic zone, [*D*] = [*D*]*_ep_*, [*D*]*_ep_* is elastoplastic matrix, [*C*] = [*C*]*_ep_*. [*C*] is a temperature-dependent vector.

A unit in the SCLP is taken. At time *t* and *t* + *dt*, the temperature are *T* and *T* + *dT*, the external force of the node are {*F^e^*} and {*f* + *dF*}*^e^*, the displacement of the node are {*u*} and {*u* + *du*}, the strain are {*ε*} and {*ε* + *dε*}, and the stress are {*σ*} and {*σ* + *dσ*}, respectively. According to the principle of virtual work, the equation can be expressed as:

(27) $${\left\{du\right\}}^{T}{\left\{F+dF\right\}}^{e}={\left\{du\right\}}^{T}{\iint_{\Delta V}\left[B\right]}^{T}\left(\left\{\sigma \right\}+\left[D\right]\left\{d\varepsilon \right\}-\left[C\right]dT\right)dV\;$$

The equation of the SCLP at time t can be expressed as:

(30) $${\left\{dF\right\}}^{e}+{\left\{dR\right\}}^{e}={\left[K\right]}^{e}\left\{du\right\}\;$$

where {*dR*}*^e^* is the force of equivalent nodal, and [*K*]*^e^* is stiffness matrix of the unit. According to elastic or plastic state of the unit, the corresponding formula is chosen to bring it into Equation (30), and simplified equation can be expressed as:

(31) $$\left[K\right]\left\{du\right\}=\sum {\left\{dR\right\}}^{e}$$

Using the Newton-Raphson method to linearize equation, the finite element equation of laser bending the SCLP can be expressed as:

(32) $$\left[K\right]\left\{\Delta u\right\}=\left\{F^{a}\right\}-\left[F^{nr}\right]$$

where [*K*] is tangent stiffness matrix, {Δ*u*} is displacement increment of the node, {*F^a^*} is the vector of external force, and [*F^nr^*] is the vector of restoring force.

## 4. Results and Discussions

### 4.1. Simulation Results and Experimental Verification of Bending Angle

**Figure 7:**
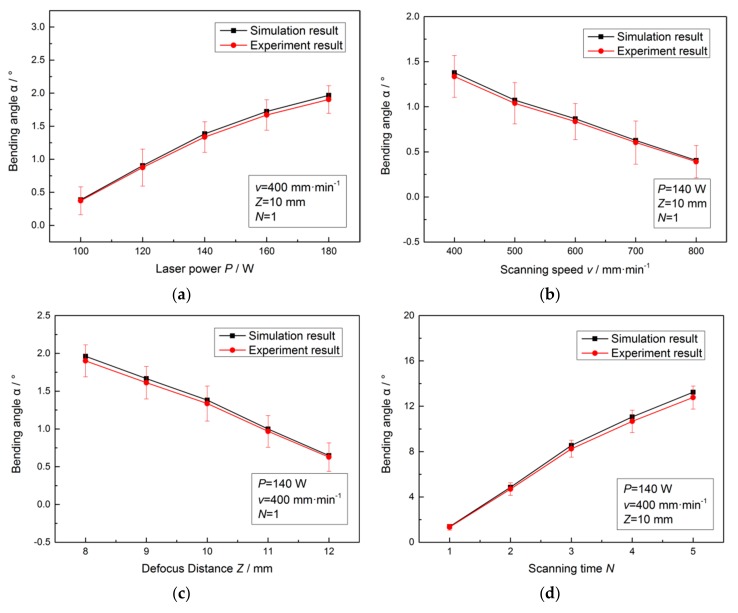
Comparison between the simulation results and the experimental verification: (**a**) Bending angle under laser power; (**b**) Bending angle under scanning speed; (**c**) Bending angle under defocus distance; (**d**) Bending angle under scanning time.

By using the equivalent method, the model of the material properties of the SCLP containing the transition layer is established. The displacement field (*h*_1_ and *h*_2_ in Equation (1)) of the SCLP is simulated under the laser loading condition by ANSYS software to predict the bending angle. At the same time, the actual bending angle is obtained by coordinate measurement. Figure 7 shows the comparison between the simulation results and the experimental verification.

Figure 7 is the bending angle of the SCLP under different laser power, scanning speed, defocus distance and scanning time. Figure 7a illustrates that the temperature gradient in the thickness direction of the SCLP increases with the increase of laser power, which leads to the extension of the plastic zone. Due to the steepening vertical strain gradient, the bending angle becomes larger. Figure 7b indicates that the increase in scanning speed produces two consequences: reduction of laser heating duration and overlap rate of laser spot. The decrease in energy density decreases the temperature gradient and thermal stress, resulting in decrease of the bending angle. Figure 7c demonstrates that the energy density at the center of the laser heating zone decreases with the increase of defocus distance, which leads to temperature gradient and plastic strain gradient decrease, and subsequent bending angle decrease. Figure 7d shows that the superposition of scanning time prolongs the processing time. Therefore, the bending angle produced by each scan is obviously increased after superposition.

**Figure 8:**
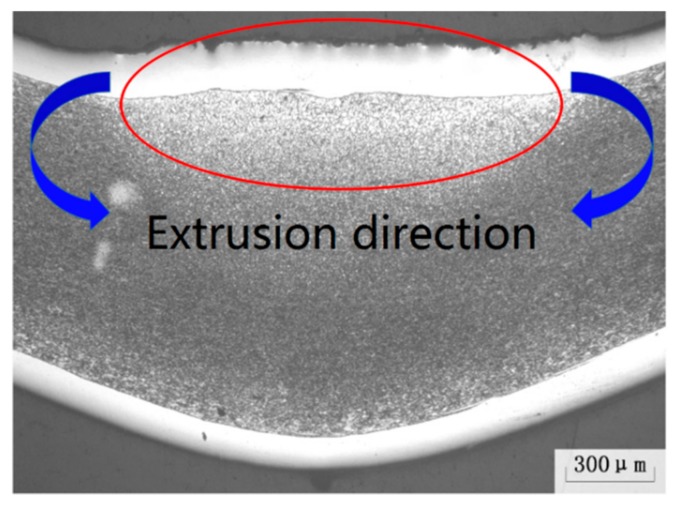
The thickening phenomenon of the SCLP.

In addition, it is found from Figure 7 that the simulation results of the bending angle based on the equivalent method are slightly larger than that of the experimental verification. Merklein et al. [15] summed up the possible reasons for a clearly dislocation phenomenon in the heat-affected zone of materials, which released the elastic potential energy stored during the bending process and reduced the bending angle. In addition, there is a material thickening phenomenon on the upper surface of the SCLP shown in Figure 8. The direction of stress generated by material accumulation is opposite to the bending direction. Due to this counteraction, the actual bending angle is smaller than the model value.

### 4.2. Comparison of Bending Angles between Equivalent Method and Mean Value Method

The bending angle of simulations and measurements are shown in Table 4, Table 5, Table 6 and Table 7. Both of the simulation methods are compared with the experimental results as follows:

(33) $$\delta_{1}\;=\;\left(\beta_{1}-\beta_{3}\right)/\beta_{3}\;$$

(34) $$\delta_{2}\;=\;\left(\beta_{2}-\beta_{3}\right)/\beta_{3}\;$$

where *β*_1_ and *β*_2_ are the bending angles of the mean value method and the equivalent method, respectively; *β*_3_ is the bending angle of experiment; *δ*_1_ and *δ*_2_ are the errors of the mean value method and the equivalent method between the simulation and experiment, respectively.

In the temperature gradient theory, Vollertsen [16] proposed a typical two-layer model to describe the relationship between the bending angle and process parameters as follows:

(35) $$\beta =3\alpha PA/\rho Cvh^{2}$$

where *β* is bending angle, *α* is thermal expansion coefficient, *P* is laser power, *A* is absorption coefficient, *ρ* is density, *C* is specific heat capacity, *ν* is scanning speed and *h* is the thickness of the plate.

It can be seen from the formula that the correlative properties of materials include thermal expansion coefficient, density and specific heat capacity. Comparing material properties with the mean value method and the equivalent method can be seen from Table 3:

(36) $$\alpha_{m}=1.01\alpha_{c}\;$$

(37) $$\rho_{m}=1.00\rho_{c}\;$$

(38) $$C_{m}=0.99C_{c}\;$$

where *α*_m_, *ρ*_m_ and *C*_m_ are thermal expansion coefficient, density and specific heat capacity with mean value method, respectively. The Equations (36)–(38) are substituted into Equation (35) to obtain the relationship between the two bending angles as follows:

(39) $$\beta_{1}\;=\;1.02\beta_{2}$$

where *β*_1_ and *β*_2_ are bending angles of the mean value method and the equivalent method, respectively.

Compared to the mean value method, thermal expansion coefficient of equivalent calculation is lower, but specific heat capacity is higher. Besides, the material density of the two methods is basically equal. As the specific heat capacity increases, the bending angle decreases. As thermal expansion coefficient decreases, the bending angle decreases. According to Vollertsen’s formula, under the combined effect of the three parameters, the bending angle based on the equivalent method is smaller than that of the mean value method for the transition layer. There is a positive correlation between the bending angle of the transition layer and the SCLP, which explains that the bending angle using the equivalent properties is less than the bending angle using average properties in the simulation.

Under the condition of different parameters, the maximum of bending angle errors with two simulations is 4.93% and 3.74%, respectively. The accuracy of the equivalent method is improved by 1.19% than the mean value method. Therefore, the equivalent method is more effective than the mean value for calculating the properties of the transition layer to improve the accuracy of simulation in the SCLP laser bending process with a better prediction of bending angle.

## 5. Conclusions

In this study, a 7 μm thick transition layer is formed between the stainless steel layer and carbon steel layer by the interdiffusion of elements due to the concentration gradient. The electron micro probe analysis found that the transition layer is composed of 46.63% stainless steel and 53.37% carbon steel with volume friction. Based on the equivalent calculation of material properties in the transition layer, the simulation of the laser bending process is carried on to predict the bending angle of the SCLP. The main conclusions are summarized as follows:

1. Based on the element distribution in the SCLP as well as the volume friction of the stainless steel and the carbon steel in the transition layer, the properties of the transition layer, including equivalent thermal conductivity, equivalent thermal expansion coefficient, equivalent elastic modulus, equivalent density, equivalent Poisson’s ratio, and equivalent specific heat capacity, are calculated by the equivalent method. The values of these properties are 35.22 W·m^−1^·K^−1^, 1.41 × 10^−5^ K^−1^, 2.06 × 10^11^ Pa, 7892.64 kg·m^−3^, 0.287, 631.15 J·kg^−1^·K^−1^, respectively.
2. A finite element model of the laser bending SCLP is developed based on the thickness and equivalent properties of the transition layer. The bending angle of the SCLP increases with the increase in laser power and scanning time, and decreases with the increase in scanning speed and defocus distance.
3. At the same process parameters, the simulation results are in good agreement with the experimental data, with maximum 3.74% of errors. The simulation results of the modifying finite element model are compared with the results of the mean value method; the accuracy of the simulation results is improved by 1.19%.

## Figures and Tables

**Table 1:** Experiment conditions with different laser parameters.

Classification	Process Parameters	Unit	Value Ranges
Processing parameters	Power *P*	W	80, 100, 120, 140
	Scanning speed *v*	mm·min^−1^	400, 600, 800, 1000
	Defocus distance *Z*	Mm	+10, +11, +12, +13
	Scanning number *N*		1, 2, 3, 4
Laser parameters	Laser frequency *f*	Hz	40
	Pulsed width *t*_p_	Ms	2

**Table 2:** Energy dispersive spectrometer analysis.

Measuring Position	Element Content (%)			
	Fe	Cr	Ni	C and Others
Stainless steel	70.24	17.66	9.63	2.47
Transition layer	85.02	8.63	5.18	1.17
Carbon steel	97.64	0.11	0.09	2.16

**Table 3:** Material properties of the SCLP.

Property	Unit	Stainless Steel	Transition Layer		Carbon Steel
			Equivalent Method	Mean Value Method	
Thermal conductivity *k*	W·m^−1^·K^−1^	15.80	35.22	38.45	61.10
Heat expansion *α*	K^−1^	1.66 × 10^−5^	1.41 × 10^−5^	1.43 × 10^−5^	1.20 × 10^−5^
Specific heat capacity *C*	J·kg^−1^·K^−1^	502.00	631.15	623.50	745.00
Density *ρ*	Kg·m^−3^	7930.00	7892.64	7895.00	7860.00
Poisson’s ratio *λ*		0.2850	0.2866	0.2865	0.2880
Young’s modulus *E*	Pa	2.00 × 10^11^	2.06 × 10^11^	2.06 × 10^11^	2.12 × 10^11^

**Table 4:** Comparison of bending angle and error between the mean value method and the equivalent method with different power.

*P*/W	*v* = 400 mm·min^−1^	*Z* = +10 mm	*N* = 1	*δ*_1_	*δ*_2_
	*β*_1_ (Mean Value)	*β*_2_ (Equivalent)	*β*_3_ (Experiment)		
100	0.391	0.386	0.373	4.83%	3.49%
120	0.917	0.903	0.875	4.80%	3.20%
140	1.392	1.381	1.336	4.19%	3.37%
160	1.746	1.724	1.671	4.49%	3.17%
180	1.983	1.967	1.904	4.15%	3.31%

**Table 5:** Comparison of bending angle and error between the mean value method and the equivalent method with different scanning speed.

*v*/mm·min^−1^	*P* = 140 W	*Z* = +10 mm	*N* = 1	*δ*_1_	*δ*_2_
	*β*_1_ (Mean Value)	*β*_2_ (Equivalent)	*β*_3_ (Experiment)		
400	1.392	1.381	1.336	4.19%	3.37%
500	1.085	1.073	1.039	4.43%	3.27%
600	0.878	0.867	0.837	4.90%	3.58%
700	0.632	0.624	0.604	4.64%	3.31%
800	0.410	0.405	0.391	4.86%	3.58%

**Table 6:** Comparison of bending angle and error between the mean value method and the equivalent method with different defocus distance.

*Z*/mm	*P* = 140 W	*v* = 400 mm·min^−1^	*N* = 1	*δ*_1_	*δ*_2_
	*β*_1_ (Mean Value)	*β*_2_ (Equivalent)	*β*_3_ (Experiment)		
+8	1.985	1.961	1.901	4.42%	3.16%
+9	1.688	1.667	1.612	4.71%	3.41%
+10	1.392	1.381	1.336	4.19%	3.37%
+11	1.010	0.999	0.968	4.34%	3.20%
+12	0.653	0.647	0.627	4.15%	3.19%

**Table 7:** Comparison of bending angle and error between the mean value method and the equivalent method with different scanning time.

N	*P* = 140 W	*v* = 400 mm·min^−1^	*Z* = +10 mm	*δ*_1_	*δ*_2_
	*β*_1_ (Mean Value)	*β*_2_ (Equivalent)	*β*_3_ (Experiment)		
1	1.392	1.381	1.336	4.19%	3.37%
2	4.916	4.861	4.695	4.71%	3.54%
3	8.643	8.545	8.247	4.80%	3.61%
4	11.187	11.062	10.671	4.84%	3.66%
5	13.406	13.254	12.776	4.93%	3.74%
